# Evolution of male genitalia: environmental and genetic factors affect genital morphology in two *Drosophila *sibling species and their hybrids

**DOI:** 10.1186/1471-2148-7-77

**Published:** 2007-05-15

**Authors:** Ignacio M Soto, Valeria P Carreira, Juan J Fanara, Esteban Hasson

**Affiliations:** 1 Departamento de Ecología, Genética y Evolución, Facultad de Ciencias Exactas y Naturales, Universidad de Buenos Aires, Buenos Aires, Argentina

## Abstract

**Background:**

The rapid evolution of genital morphology is a fascinating feature that accompanies many speciation events. However, the underlying patterns and explanatory processes remain to be settled. In this work we investigate the patterns of intraspecific variation and interspecific divergence in male genitalic morphology (size and shape) in the cactophilic sibling species *Drosophila buzzatii *and *D. koepferae*. Genital morphology in interspecific hybrids was examined and compared to the corresponding parental lines.

**Results:**

Despite of being siblings, *D. buzzatii *and *D. koepferae *showed contrasting patterns of genital morphological variation. Though genitalic size and shape variation have a significant genetic component in both species, shape varied across host cacti only in *D. buzzatii*. Such plastic expression of genital shape is the first evidence of the effect of rearing substrate on genitalic morphology in *Drosophila*. Hybrid genital morphology was not intermediate between parental species and the morphological resemblance to parental strains was cross-dependent.

**Conclusion:**

Our results suggest the evolution of different developmental networks after interspecific divergence and the existence of a complex genetic architecture, involving genetic factors with major effects affecting genital morphology.

## Background

The evolutionary processes governing the divergence of animal genitalia are mostly unknown and constitute one of the most intriguing pieces of a mayor puzzle that is speciation [1-5]. In many arthropod groups male genitalia evolves at particularly high rates and this special feature constitutes the mechanistic basis of its use as a specific diagnostic trait [6,7]. Notwithstanding the taxonomic importance of genitalic morphology, intraspecific studies addressing the causes and consequences of intraspecific morphological variation are scarce. Such studies, using methods successfully applied in evolutionary biology, offer the opportunity to gain new insights into the evolutionary processes and forces involved in genitalic evolution [3].

Three main hypotheses have been proposed to explain the evolution of genital morphology: the lock and key, the pleiotropy and the sexual selection hypotheses. The lock and key hypothesis [8] states that male genitalia evolve as a species-specific trait in order to properly fit in female genitalic organs. This theory predicts a canalized development of male genitalia and low levels of phenotypic and genotypic variation, since genitalic traits are expected to be under strong stabilizing selection [3,9].

The pleiotropy hypothesis assumes that genital variation is largely neutral. Since genital and non-genital morphological traits are implicitly genetically correlated, changes of allele frequencies at loci pleiotropically affecting general morphology and genitalia may lead to rapid and arbitrary evolution of genitalic traits [2,10,11]. The sexual selection hypothesis states that morphological differences in male genitalia are related to variation in fertilization success and that morphological divergence is driven by sexual selection [3].

The study of the evolution of male genitalia may be even more complicated not only because it may be influenced by both natural and sexual selection, but also because it's phenotypic expression might be influenced by environmental factors [12] as occurs for other morphological traits. Thus, the joint study of intraspecific variation and interspecific divergence may provide a useful approach for the understanding of the underlying genetic architecture of genital traits and the evolutionary processes involved [13-15]. In this sense, it has been suggested that differences in genital morphology between closely related species would be largely polygenic [16]. Such claim is based on the single study comparing the morphology of male posterior lobe in two closely related species of *Drosophila *and their hybrids [17]. Therefore, it is clear that more studies are necessary to further support this affirmation and to determine whether simple genetic differences can account for the evolution of fast evolving and complex structures such as male genitalia.

The aedeagus, which is the intromittent organ of male genitalia [18], is considered the main diagnostic trait for species recognition in the *Drosophila repleta *group [6]. To this group belong the South American *D. buzzatii *and *D. koepferae *[19,20], which are morphologically undistinguishable except for conspicuous differences in male genitalia. These species are reproductively isolated by partial ecological isolation [21], sexual isolation and post mating barriers [22]. Both species can breed and feed on the necrotic tissues of several cacti species [23,24], however they exhibit a certain degree of niche separation; *D. buzzatii *is mainly adapted to breed on decaying tunas (genus Opuntia), while *D. koepferae *prefers the necrotic stems of columnar cacti of the genera *Cereus *and *Trichocereus *[21]. Though sexual isolation between these species is strong, behavioral barriers can be forced in the laboratory, since *D. buzzatii *males can inseminate *D. koepferae *females and female hybrid offspring can be successfully backcrossed to *D. buzzatii *males [25]. Furthermore, recent population genetic studies have provided indirect evidence of past or recent gene flow between these species [26,27].

The knowledge of the ecology of these cactophilic *Drosophila *[21,24,28,29], coupled with the possibility to produce interspecific hybrids in the laboratory and the potential for natural hybridization, makes this pair of species into an excellent model for speciation studies, particularly those addressing the genetic and ecological basis of morphological change associated to interspecific divergence.

In this work we investigate the sources of phenotypic variation, genetic and environmental, by examining genitalic size and shape in flies of several isofemale lines of both species and interspecific hybrids raised in two different species of host cacti.

## Results

A total of 606 males were analyzed in this study (252 *D. buzzatii*, 294 *D. koepferae *and 60 interspecific hybrids).

**Figure 1:**
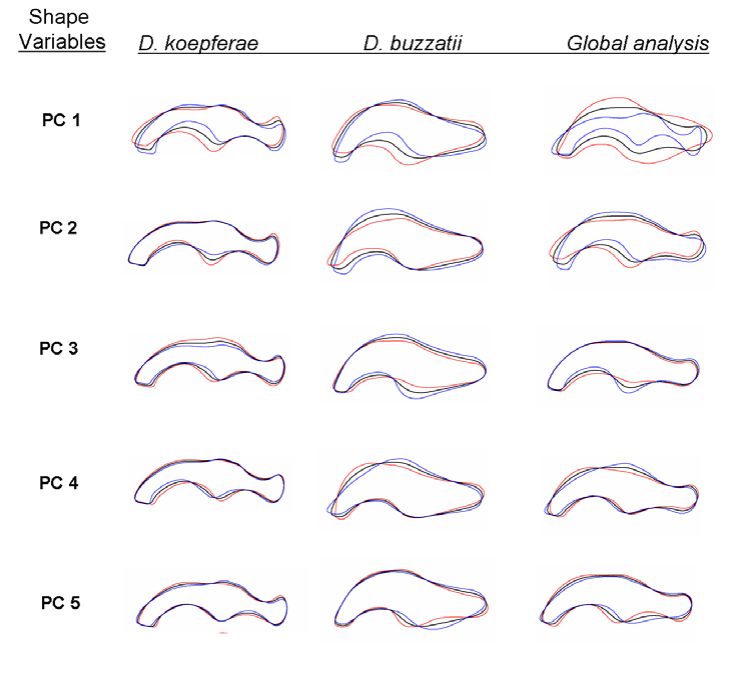
**Shape variation**. Outlines represent the variation in shape accounted by the first five principal components. Each outline was reconstructed from the estimated coefficients by letting the score of the corresponding principal component be equal to the mean and mean plus or minus two standard deviations (SD) and the remaining components set to zero. Black line, blue line and red line stand for mean shape, mean + 2 SD and mean - 2 SD respectively.

The total number of principal components explaining a significant proportion of shape variation was 13 in *D. koepferae *and 12 in *D buzzatii *(results not shown). The cumulative contribution of the first 5 principal components of the elliptic Fourier descriptors (EFDs) of the genital outlines accounted for over 74% and 77% of total shape variance in *D. koepferae *and *D. buzzatii*, respectively and nearly 84% of shape variation in the interspecific analysis (Table 1). The proportion of morphological variation summarized by each PC is illustrated in Figure 1.

**Table 1:** Shape variation.

	**Intraespecific analyses**				**Interespecific analysis**	
	*D. koepferae*		*D. buzzatii*		Parental species + hybrids	

**Shape variables**	Eigenvalue	Proportion(%)	Eigenvalue	Proportion(%)	Eigenvalue	Proportion(%)

PC1	1.94E-03	36.6	2.63E-03	30.3	6.26E-03	50.4
PC2	8.95E-04	16.8	1.57E-03	18.1	2.02E-03	16.3
PC3	4.85E-04	9.1	1.21E-03	13.9	1.05E-03	8.4
PC4	3.70E-04	7	8.16E-04	9.4	5.57E-04	4.5
PC5	2.50E-04	4.7	5.22E-04	6	4.97E-04	4

Percent of genital morphological variance explained by the first 5 principal components (PCs) in intraspecific and global (*Dorosphila buzzattii *+ *D. Koepferace *+ hybrids) analyses.

### Parental species size and shape variation

We detected significant differences in aedeagus size between species but more notably between flies reared in different cacti (Table 2a). However, in a nested ANOVA design as ours, the random factors Line in Species and Line by Cactus are the error terms of the fixed factors (Species, Cactus and their interaction). Large values of these terms may provide unreliable results in the testing of the fixed factors. Consequently we performed "a posteriori" contrasts of the Species by Cacti interaction to confirm the general results. Post hoc comparisons showed that *D. buzzatii *male flies reared in *Opuntia *had larger male genitalia than those grown in *T. candicans *(Tukey, *p *= 0.028) while in *D. koepferae *differences between flies grown in different cactus media were not significant (Tukey, *p *= 0.69). As visually observed, *D. buzzatii *and *D. koepferae *significantly differed in their genitalic shape and presented morphological variation not only among lines within species but also interacting with the breeding substrate (Table 2a).

**Table 2:** Sources of variation of male genitalia in *Drosophila buzzatii *and *D. koepferae*.

**Sources of variation**	**df**	**Size**	**Shape**				
			PC1	PC2	PC3	PC4	PC5

a)							
Species (Sp)	1	1812.95*	3519.85*	3.39	0.15	0.11	5.07*
Cactus	1	6.56*	0.04	0.95	1.47	0.39	2.62
Species × Cactus	1	0.77	0.53	0.02	0.99	1.42	1.18
Line(Sp)	28	2.05*	5.91*	2.36*	3.66*	3.08*	1.75
Line(Sp)*Cactus	28	1.99*	1.33	1.72*	0.99	1.88*	1.74*
Error	487						
							
b)							
*Drosophila koepferae*							
Line	14	4.71*	6.99*	4.35*	3.97*	3.29*	2.63*
Cactus	1	4.02	0.62	0.24	0.5	0.49	0.23
Line × Cactus	14	1.12	0.89	1.1	2.00*	1.41	1.35
Error	264						
							
*Drosophila buzzatii*							
Line	14	1.39	3.25*	3.71*	4.08*	6.87*	3.65*
Cactus	1	3.31	0.07	1.67	2.71	1.88	0.04
Line × Cactus	14	2.47*	2.18*	1.1	1.06	1.25	1.33
Error	224						

*F *values of the ANOVAs and MANOVAs for aedeagus size and shape respectively in interspecific (a) and intraspecific (b) analyses. * *p *< 0.01

The results of intraspecific ANOVAs also revealed important differences (Table 2b). In *D. buzzatii *the Cactus by Line interaction was significant and accounted for a relatively high percentage (12.1%) of phenotypic variance in aedeagus size. However, only the Line factor was significant in *D. koepferae*, neither the Cactus effect nor the Cactus by Line interaction were significant.

In summary, according to our experimental design, based on the isofemale line technique [30], shows that aedeagus size is not only phenotypically plastic, but also that substantial heterogeneity exists among lines in their plastic response, suggesting that plasticity has a genetic basis in *D. buzzatii*. In *D. koepferae*, in contrast, our results show that variation in aedeagus size has a genetic component, devoid of any plastic response in relation to the breeding substrate. According to the results of the MANOVAs, variability among lines in aedeagus shape was significant in both species (Table 2a, b). The proportion of total shape variation explained by the interaction Cactus by Line also differed between species. Approximately 9% of total shape variance was explained by the Cactus by Line interaction in *D. koepferae*. This interaction was significant for PC3 which is related with variation in thickness in both dorsal and ventral median portions of the organ (Figure 1). Conversely, in *D. buzzatii*, the Cactus by Line interaction was significant for PC1, which describes changes in the process of the ventral margin (Figure 1) and accounts for an important proportion (30%) of the explained morphological variance.

### Allometric patterns

Correlation analysis between aedeagus size and shape also revealed important interspecific differences. On one hand, aedeagus shape was strongly correlated with size in *D. buzzatii *(more than 16% of the total shape variation was allometric, Table 3). Conversely, none of the 5 principal shape variables in *D. koepferae *were significantly associated with genital size (Table 3).

**Table 3:** Within organ allometry. Correlation coefficients between size and each one of the principal components (PC) scores accounting for shape variation in the aedeagus of *D. koepferae*, *D. buzzatii *and F1 hybrids.

**Shape variables**	**Genital size**		
	*D. koepferae*	*D. buzzatii*	F1 Hybrids

PC1	-0.08	-0.17*	0.61*
PC2	-0.07	0.45*	-0.21
PC3	-0.03	0.24*	0.1
PC4	0.02	0.16*	0.14
PC5	0.03	0.01	-0.02
Total allometric shape variance (%)	0.0	16.6	30.7

Allometric shape variance was calculated as the sum of partial allometric variance of each PC significantly correlated with size. * *p *< 0.05.

We also studied the relationship between variables describing size of male genitalia and wing length. These variables were not significantly correlated in *D. koepferae *(r = 0.13, p = 0.07), whereas in *D. buzzatii*, we detected a significant allometric relationship (r = 0.32, p < 0.001). Furthermore, aedeagus size and wing length varied isometrically in *D. buzzatii *as suggested by a coefficient of allometry not significantly different from 1 (slope value of linear adjusted function = 0.82; 95% confidence interval values [0.42 to 1.23]).

### Aedeagus morphology in interspecific hybrids

Four interspecific crosses, out of 25 attempted, (crosses 4855, 4853, 8832 and 3512) yielded enough hybrid progeny as to perform the present study. These results are in agreement with previous studies reporting strong premating isolation between *D. buzzatii *and *D. koepferae *[31]. In order to asses hybrid male fertility, and prior to dissection, hybrid males were aged for 1 week and placed for 5 days in vials with several mature virgin females of *D. buzzatii *or *D. koepferae*. In all cases hybrid males failed to produce offspring even though copulation attempts were observed in the vials. Hybrid progeny obtained in crosses 4853 and 8832 could only be tested in vials prepared with the medium prepared with fermenting *Opuntia *due to low numbers of hybrid larvae, while in the other crosses the yield of hybrid progeny was high enough to be reared in both cactus media.

**Figure 2:**
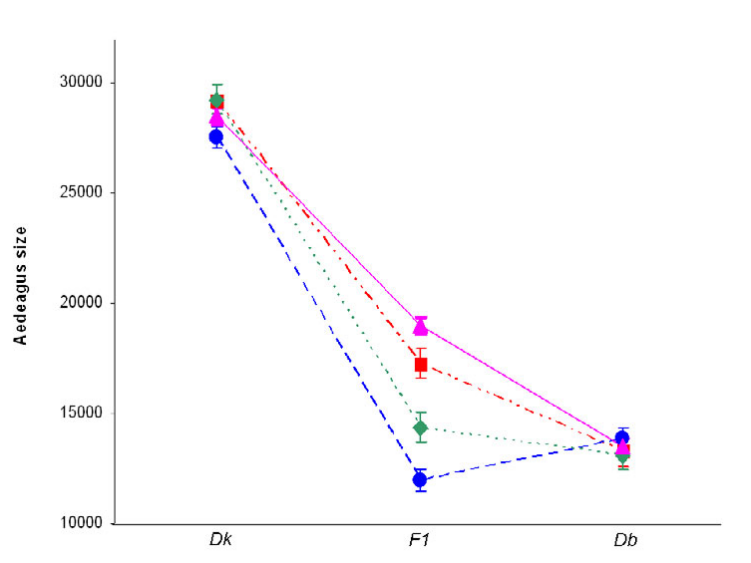
**Hybrid genital size**. Mean area size (in pixels) of aedeagus of individuals reared in vials with fermenting *Opuntia*. *Dk*: *Drosophila koepferae*; *Db*: *Drosophila buzzatii*. Cross 3512 (●); Cross 4853 (■); Cross 4855 (◆); Cross 8832 (▲).

Regarding size, differences among genotypes (hybrids plus both parental lines) were significant in all crosses (Table 4). In Figure 2 we illustrate size differences among crosses and genotypes reared in *Opuntia*, the rearing substrate where all crosses were able to be tested. F1 hybrid males from crosses 4853 and 8832 reared in *Opuntia *vials presented intermediate values that differed significantly from both parental strains (p < 0.05 in both cases, Tukey's post hoc comparisons). In the other crosses, in which hybrids could be reared in both cacti, a significant Genotype by Cactus interaction (*F*_2,69 _= 4, 49, *p *< 0.01) was only detected in cross 4855. Hybrids male progeny in this cross presented larger aedeagi than males of the parental *D. buzzatii *line in *Opuntia*, while differences between hybrids and the *D. buzzatii *parent were not significant in *Trichocereus*. In all cases, *D. koepferae *presented the largest genitalia in both cacti. In one cross (3512) mean genitalic size in hybrids was significantly lower than the male parental *D. buzzatii *line (p = 0.025, Tukey's test). Based on the correlation matrix, only PC1 scores were correlated with organ size in hybrids accounting for 50.4% of shape variation (Table 3). Unfortunately, the low number of hybrids and the high proportion of individuals with improperly unfolded wings precluded the analysis of allometric relationships between wing size and the variable describing aedeagus size.

**Table 4:** Sources of variation of male genitalia interspecific crosses.

**Sources of variation**	**Crosses**							
	*3512*		*4855*		*8832*		*4853*	

*Size*								

	*df*	*F*	*df*	*F*	*df*	*F*	*df*	*F*

Genotype	2	359.96**	2	204.17**	2	609.06**	2	176.73**
Cactus	1	0.33	1	0.41		-		-
Genotype × Cactus	2	0.14	2	4.49*		-		-
Error	38		69		56		34	
								
*Shape*								

	*df*	λ	*df*	λ	*df*	λ	*df*	λ

Genotype	20	0.015**	20	0.095**	20	0.015**	20	0.014**
Cactus	10	0.865	10	0.914		-		-
Genotype × Cactus	20	0.635	20	0.672		-		-

*F *values of the ANOVAs and Wilk's lambda of MANOVAs for aedeagus size and shape respectively. *p < 0.05; ** *p *< 0.01

Significant shape differences among genotypes were detected in all crosses (Table 4). Post hoc comparisons showed that all genotypes differed from each other in the shape of the genitalia at least in PC1 shape scores (*p *< 0.001 in all cases). However, neither the Cactus factor nor the Cactus by Genotype interaction were significant in the crosses in which hybrids were raised in the two cactus media (4855 and 3512).

**Figure 3:**
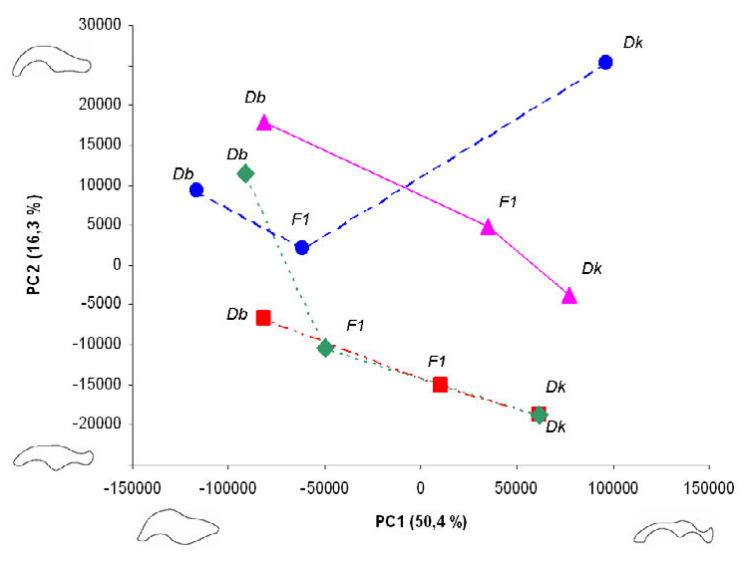
**Hybrid genital shape**. Bivariate plot of mean shape scores of each genotype for the first two principal dimensions of shape variation (and percent of variance explained). The lines drawn connect genotypes involved in the same cross. Outlines below the graphic depict the genital shape variation accounted for the first principal component. Outlines to the left of the plot represent the genital shape variation accounted for the second principal component. *Dk*: *Drosophila koepferae*; *Db*: *Drosophila buzzatii, F1*: hybrids. Cross 3512 (●); Cross 4853 (■); Cross 4855 (◆); Cross 8832 (▲).

In figure 3 we present a plot of the first two principal components describing shape variation (PC1 and PC2). The species differentiate themselves along the first shape axis (PC1) and the hybrid scores fall within the parental values. As can be observed the mean PC1 values of *D. buzzatii *lines involved in successful interspecific crosses tended to be negative and those of *D. koepferae *positive. However, hybrids failed to present intermediate values for both shape variables simultaneously. For instance, hybrids of cross 8832 had shape scores for both PC1 and PC2 that placed them in the morphological space closer to the *D. koepferae *parent (Line 88) than to *D. buzzatii *(Line 32). On the contrary, in cross 3512 a hybrid genital morphology was more similar to *D. buzzatii *for PC1 (Line12) but the mean for PC2 was more extreme than any of the parental lines. Suggestively, as explained above, hybrids in 3512 also presented smaller genitalia than both parental lines.

**Figure 4:**
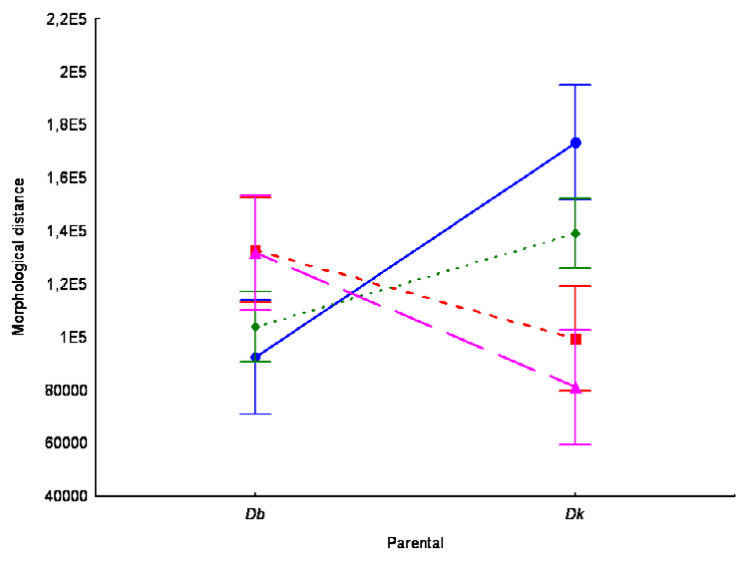
**Analysis of the morphological resemblance of hybrids to their corresponding parental strains**. Hybrid mean Euclidean distances (and confidence intervals) to each mean parental shape of each cross. *Dk*: *Drosophila koepferae*; *Db*: *Drosophila buzzatii*. Cross 3512 (●); Cross 4853 (■); Cross 4855 (◆); Cross 8832 (▲).

In order to evaluate the degree of resemblance of the morphology of hybrids to each parental line, we calculated the Euclidean distance to the morphological centroid of each parental strain using the shape (PCs) scores of each individual hybrid. As a rough index of morphological dissimilarity, hybrids would show equal mean distances to the centroids of both parental clouds of points if they have intermediate aedeagus morphology. Expression dominance of one genome over the other would produce phenotypes resembling more closely one parental strain or the other. Morphological dominant expression was tested with an ANOVA in which the variable was the Euclidean distance of each hybrid male to the centroid of each parental species (mean parental shape) with Cross and Parents as fixed factors. The ANOVA revealed significant differences among crosses though it should be noted that hybrid resemblance to parental strains were not independent of the cross (*F*_3,110_*=*17.59; *p *< 0.0001; Figure 4).

## Discussion

Aedeagus morphology is a diagnostic trait that along with chromosomal inversions provides a guide for species identification in the genus *Drosophila *and particularly in the *D. repleta *species group [6,19,20,32]. Several studies have, recently, turned the attention to the *D. buzzatii *cluster, a guild of cactophilic flies, in active cladogenesis, that inhabit the arid regions of Southern South America [33]. However, recent molecular phylogenetic studies [34] cast doubts on the reliability of male genital (aedeagus) morphology to infer the evolutionary relationships in the *D. buzzatii *cluster [33]. For instance, mitochondrial DNA sequence data place *D. koepferae *as the sister species of *D. buzzatii*, albeit the comparative analysis of aedeagus morphology indicates a close relationship with *D. serido *and allied species [34]. These results indicate that rates of evolution of male genitalia may be heterogeneous among branches of the clusters' phylogenetic tree. In this context, our study, motivated by such inconsistency between molecular and morphological data, provides valuable information that could help to distinguish the relevant factors involved in morphological variation of male genitalia in *D. buzzatii *and *D. koepferae*, a pair of species in the heart of an evolutionary conflict.

The first issue raised by our study is that aedeagus size and shape vary substantially in both species, and, that a significant portion of variation is genetically determined. Moreover, the inclusion, in our experimental design, of semi-natural rearing media prepared with different cactus hosts permitted the characterization of morphological variation in terms of phenotypic plasticity in both species. In this sense, phenotypic plasticity in genital morphology was evident in *D. buzzatii*: flies emerged in alternative cactus hosts showed significant differences in aedeagus size and shape. Moreover, the analysis of the sources of variation also revealed a significant Line by Cactus interaction in *D. buzzatii*, i.e. isofemale lines did not respond in the same way to the two environments tested, suggesting that the plastic response of genital morphology has a genetic basis [35]. Such a plastic expression of genital morphology constitutes the first evidence of a rearing substrate affecting genital morphology in *Drosophila*, and is in line with a recent study showing a plastic response of aedeagus morphology in relation to rearing temperature in *D. mediopunctata *[12]. In contrast, comparisons between males of *D. koepferae *emerged in different cactus media did not reveal any sign of phenotypic plasticity, suggesting a more canalized (non plastic) development of male genitalia.

Another relevant point is that patterns of allometry within (aedeagus size and shape) and between organs (aedeagus and wing size) also differed between *D. buzzatii *and *D. koepferae*. In the latter, shape and size of male genitalia, as well as aedeagus size and wing length, appeared to be largely uncoupled as suggested by the low level of within and between organ allometries. These results suggest that differences among flies in wing length are not expected to be accompanied by changes in the size of the genitalia, indicating that the factors involved in development of wings and male genitalia are largely independent in *D. koepferae*. In contrast, aedeagus shape and size were significantly correlated in *D. buzzatii*, suggesting that factors affecting size (for instance the type of cactus host) may also indirectly affect the shape of the organ.

In *D. buzzatii*, body size related traits (such as wing length), known to be under natural selection [36-38], are affected by the nature of the breeding substrate [21,39-41]. Furthermore, a positive correlation between body size and male reproductive success is well known in this species [38,42-44]. If the phenotypic correlation observed between aedeagus size and wing length has a genetic basis, any directional selective pressure affecting body size (wing length) would indirectly affect the evolution of male genitalia.

Several features of the mating system, such as female remating frequency, premating time, copulation duration, interval between successive matings, and progeny numbers, have been shown to be genetically variable in *D. buzzatii *[45]. However, the connection between these traits and male genital morphology has not been explored. Indeed, the implications of our present results in genital evolution and speciation would obviously depend on the kind of relationship between genital morphology and mating success, as it was reported to occur in other insect taxa such as Heteroptera [46] and Coleoptera [15].

To this point we have presented basic features of the patterns of variation of aedeagus morphology in *D. buzzatii *and *D. koepferae*, now, we would like to examine whether our data allow a critical evaluation of the plausibility of the three main hypotheses proposed to explain genital evolution. Though our results are not entirely conclusive in this respect, the extensive phenotypic and genetic variation in aedeagus morphology are strong evidence against the "lock and key" hypothesis, that predicts low levels of variation (both phenotypic and genetic) in genital structures. However, the correlation between aedeagus morphology and wing length, along with the condition dependence (phenotypic plasticity in relation to cactus hosts) are in agreement, at least in *D. buzzatii*, with predictions of the hypothesis of pleiotropy. Concerning the third hypothesis, sexual selection, we must await for the results of experiments testing the relationship, if any, between genital morphology and reproductive performance.

The final issue we would like to address is whether our study allows us to envisage the genetic architecture underlying differences in male genitalia between *D. buzzatii *and *D. koepferae*. In this sense, our results seem in conflict with the single available work comparing genital morphology in hybrids and parental species [17,47]. Genital size and shape of hybrid males were not intermediate and the morphological resemblance of hybrids to either *D. buzzatii *or *D. koepferae *varied among crosses. In fact, hybrid's morphological distance to *D. buzzatii *and *D. koepferae *depended on the parental strains employed in the crosses. In none of the 4 crosses hybrid morphology was phenotypically intermediate (Figure 4). Hybrids from crosses 4853 and 8832 tended to be more similar to the *D. koepferae *parental strain, while in crosses 3512 and 4855 the morphology of the hybrid genitalia resembled closer that of *D. buzzatii *male parent. These results seem to be incompatible with the idea that interspecific differences in the morphology of male genitalia are caused by polygenes with small additive effects as claimed by Coyne and Orr for *D. simulans *and *D. mauritiana *[16] (however, it should be noted that the authors in [47] acknowledged some degree of dominance and epistasis). Actually, our results suggest a complex genetic architecture probably involving a certain degree of dominance and the involvement of genetic factors with large effect.

However, there are certain differences between our study and Liu et al's [47] that are worth mentioning. The first relates to the part of the genitalia examined in each case, the intromittent organ in *D. buzzatii *and *D. koepferae *and the posterior lobe (a particular element of male genitalia in the *D. melanogaster *group [48]) in *D. simulans *and *D. mauritiana*. These organs perform different functions during copulation [48] and therefore their evolution might be governed by different processes. The second is methodological and can be avoided by applying our methodology to Liu et al's dataset. To this end, we captured the outlines available in the digital version of [47], and tested for shape differences between F1 hybrids and parental species. This reanalysis confirmed that F1 hybrids have an intermediate morphology between *D. simulans *and *D. mauritiana *(the morphological distances between hybrids to both parental phenotypes were not significantly different: *F*_1, 18 _=, 008, *p *= 0, 93). Another non trivial point, that may complicate our interpretation is the difference in the time of divergence between the members of the two pairs of species, since development in interspecific hybrids is a result of a balance between the effects of the degree of heterozygosity and the degree of genomic coadaptation (expected to increase/decrease as a function of divergence, respectively) and the outcome of the past selection pressures on the species studied (see [49]). *D. simulans *and *D. mauritiana *are two recently derived species that shared their last common ancestor 0.6 – 0.9 million years ago [50], while *D. koepferae *and *D. buzzatii *are older species that diverged 5 million years ago [26]. Finally, *D. simulans *and *D. mauritiana *are homosequential species, i.e. their basic polytene chromosome banding patterns are identical [51], whereas two inversions became fixed since divergence in *D. buzzatii *and *D. koepferae*. In addition, rich second chromosome inversion polymorphisms have evolved independently in the latter pair of species [20,23]. Our knowledge of inversion polymorphisms is mostly restricted to *D. buzzatii*, in which polymorphic inversions are known to affect morphological and fitness related traits (see [52] and references therein). Although there is no direct evidence linking inversions and genital morphology, inversions may affect aedeagus morphology via its effect on general body size (recall the allometric relationship detected between aedeagus size and wing length in *D. buzzatii*). In this context, the idea that morphology (size and shape) of an organ potentially involved in species recognition (such as aedeagus morphology), might be associated to polymorphic inversions is consistent with recent theories linking chromosomal rearrangements and reproductive isolation [53,54], and deserves further investigation.

## Conclusion

Our comparative study of patterns of intraspecific variation in male genital size and shape and intra and inter-organ allometries in *D. koepferae *and *D. buzzatii *suggest different scenarios of genital evolution and probably the evolution of different developmental networks. Moreover, our study suggests that extrapolations across species are unwarranted, different evolutionary mechanisms might be involved in the evolution of genital morphology even in closely related species.

## Methods

### Experimental design

Fifteen isofemale lines (lines hereafter) of each species, derived from collections in the locality of Suyuque (San Luis province, Argentina), were employed in the experiments outlined below. In this area both species coexist in nearly equal proportions (45% of captured females were *D. koepferae*, 55% *D. buzzatii*). The advantages of the use of the isofemale line technique in quantitative evolutionary genetics have been thoroughly described in [30]. Briefly, the isofemale line technique is a convenient methodology for the analysis of quantitative traits under laboratory conditions. All experiments described below were conducted 24 generations after the foundation of isofemale lines.

Thirty first-instar larvae from each line were seeded in vials containing 6 ml. of media prepared with artificially fermented cactus (see [41] for details). Two different cactus species, *Opuntia sulphurea *and *Trichocereus candicans *which are commonly used as breeding substrates by *D. buzzatii *and *D. koepferae *in the locality sampled, were employed for the preparation of the semi-natural media. For each line, 4 replicated vials were run in each cactus type.

Five lines of each species were randomly chosen to generate interspecific hybrids. All possible combinations were attempted (25 potential crosses). F1 hybrids were produced by crossing 25 virgin females of a *D. koepferae *line with 50 males of a *D. buzzatii *line. The reciprocal cross invariably failed to produce viable progeny. Crosses were identified according to the number of the female parental line (*D. koepferae*) preceding the number of the male parental (*D. buzzatii*) line (e.g. the cross between *D. koepferae *line 48 and *D. buzzatii *line 55 was designated as 4855). Batches of 30 first-instar hybrid larvae were transferred to culture vials containing one of the two 'semi-natural' media. Four replicated vials were run per every combination of cactus and cross when the number of hatched larvae allowed it. The hybrid status of the descendants was ascertained by the cytological analysis of the polytene chromosomes of progeny larvae grown in vials run in parallel.

All cultures were maintained at 25 +/- 1°C with a 12:12 light/dark photoperiod until the emergence of adults. Adult flies were simultaneously collected and sexed under light CO_2 _anesthesia.

The aedeagus and both wings were dissected from 2 to 5 males emerged in each replicate. Aedeagi were mounted on slides and photographed with a digital camera mounted on a microscope at 400 × magnification. Wings were also mounted and ventral views of wing images were captured with a digital camera attached to a binocular microscope (25 ×) connected to a computer. In each image, we scored total wing length (WL) using TpsDig [55].

### Morphological quantification

**Figure 5:**
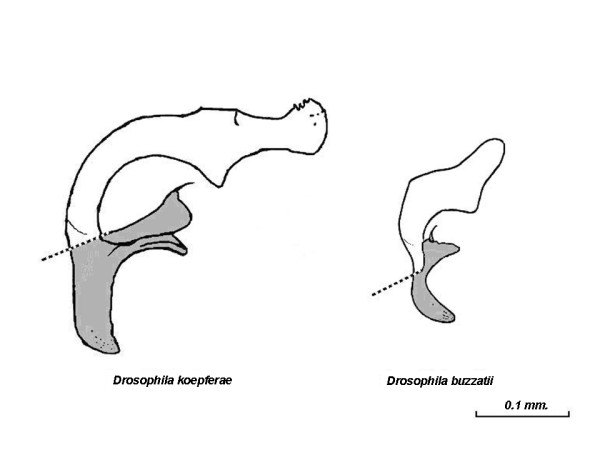
**Aedeagus morphology**. Lateral aspect of the intromittent organ in *Drosophila koepferae *and *D. buzzatii *(modified with permission from [6]). Shaded areas represent the portion excluded from the shape quantification (See [6] for details of the morphology of male genitalia).

As shown in Figure 5 there are conspicuous differences in aedeagus morphology between *D. buzzatii *and *D. koepferae *that preclude the possibility of determining an adequate number of reliable homologous landmarks. However, the aedeagus is a flat quitinous organ that can be effectively described in shape and size in two dimensions when flattened under a cover slip. Consequently, we decided to employ an approach based on elliptic Fourier descriptors (EFDs) [56] as a proper resolution to the problem of shape quantification [47]. This is a type of analysis in which differences in *x *and *y *coordinates of the outline of the studied organ are fit separately as functions of arc length by Fourier analysis, so that the outline can be decomposed into a weighted sum of sine and cosine functions designated as harmonics. Outlines from digital images were used to obtain Fourier coefficients for a polynomial function of 30th degree which were computed with SHAPE v1.2 package, [57] using Elliptic Fourier analysis [56,58,59]. For the quantification of organs' shape we only considered the distal part of the aedeagus excluding the apodeme and the gonopods (Shaded areas in Figure 1. Thus, we simplified the studied contour by taking into account only the portion of the organ effectively involved in the penetration of female genitalia.

The area of each aedeagus (in pixels) was calculated from the digital images and considered as an estimator of the size of the organ. We performed a normalization of the descriptors based on the first harmonic ellipse that corresponds to the first Fourier approximation to the contour information (reviewed in [60]). Thus, size, orientation and starting position of the contours were standardized in accordance with the size and alignment of the major axis of the first ellipse, leading to representations of the organs that are only based on internal properties of the outlines (i.e. shape) [56].

The variance-covariance matrix of the 120 (4 per harmonic) estimated EFDs coefficients was used as input in a principal components analysis. This procedure allowed us to summarize the information assessed in the coefficients and reduce the dimensionality of the variables [61] in a lower number of principal components (PC). Thus, the resulting scores of each PC of each specimen could be considered as reorganized uncorrelated morphological traits representing different aspects of total shape variation [57] that were used as shape descriptor variables in subsequent analyses. For the sake of simplicity and in order to avoid components accounting for possible biologically meaningless morphological variation only the first 5 components of both intraspecific and interspecific analyses were considered as shape variables in the ANOVAs.

We worked with two sets of PC scores. The first set of principal components were calculated from the matrix of coefficients derived form the analysis of the outlines of the genitalia of males of the parental lines employed in successful interspecific crosses and the hybrids. The second set was obtained separately for each species improving the assessment of intraspecific variation in aedeagus morphology by avoiding the noise resulting from conspicuous interspecific morphological differences in the estimation of the PCs. This set was used in the evaluation of intraspecific sources of shape variation. Preliminary analyses with this technique showed that it is repeatable and reliable in species discrimination [62].

### Analysis of variance of aedeagus size and shape

Both interspecific and intraspecific size differences were investigated by means of ANOVAs, with Species (2 levels, fixed factor), Cactus (2 levels, fixed factor) and Line (nested in Species random factor) as main sources of variation. The variable was log-transformed to ensure homoscedasticity. According to our experimental design, in the ANOVAs for species, a significant cactus effect (*C*) may be interpreted as phenotypic plasticity, while significant differences among isofemale lines as due to genetic differences (since all lines were reared under the same conditions). Finally, a significant *L × C *interaction may be construed as an estimation of genotype by environment interaction (GEI) or more explicitly, that the response of isofemale lines is not independent of the rearing cactus.

Shape variation was assessed by means of MANOVAs using the scores of each PC as dependent variables and Cactus and Line as main sources of variation.

In the presence of allometry, a fraction of the changes in shape might be explained by changes in the size of the organ. We were interested to comparatively evaluate the amount of allometric change in genital morphology in these species. Thus, we calculated Pearson's product-moment correlation matrix among shape scores and aedeagus size in each species separately to explore the relationship between aedeagus shape and general size of the aedeagus (within organ allometry).

We also evaluated the allometric relationship between aedeagus size and wing length, a trait correlated with overall body size, which in the studied species are known to be affected by the rearing substrate [41]. Size data were log-transformed prior to all analysis.

The principal components scores obtained by means of the general assessment of interspecific shape variation of parental lines and F1 hybrids were used in the examination of morphological patterns of variation in the offspring of interspecific crosses. Size was analyzed by means of an ANOVA and shape with a MANOVA. In both cases the principal factor was Genotype (both parents and the hybrids) and in those crosses in which hybrid larvae could be reared in both cactus hosts, Cactus was also considered as a fixed factor.

Statistica 6.0 [63] was used for statistical analyses and in all cases the corresponding assumptions were properly tested.

## Authors' contributions

IS and EH conceived the study and read the salivary gland squashes. IS and VC carried out the experimental crosses, egg collections, larval seeding, rearing and adult collection. IS dissected the male genitalia, performed the morphological quantification, statistical analyses, and wrote the first draft of this manuscript. IS and EH wrote the final version of this manuscript. VC mounted the wings on microscope slides and provided the wing measurements. VC and JF helped to draft the final version of this manuscript. All authors read and approved the final manuscript.
